# Oxycodone combined with pulsed radiofrequency for refractory cancer pain from spinal metastases: a randomized controlled study

**DOI:** 10.1007/s00520-026-11122-x

**Published:** 2026-08-24

**Authors:** L Li, Hj Wang, Xj Wang, Sc Xing, Jh Zhong

**Affiliations:** Department of Anesthesiology, The Affiliated Yangming Hospital of Ningbo University (Yuyao People’s Hospital), Yuyao, Zhejiang Province People’s Republic of China

**Keywords:** Refractory cancer pain, Spinal metastases, Oxycodone, Pulsed radiofrequency

## Abstract

**Objectives:**

To assess the clinical efficacy and safety of oxycodone combined with pulsed radiofrequency (PRF) in the treatment of refractory cancer pain (RCP) arising from spinal metastasis.

**Methods:**

A randomized controlled trial enrolled 60 patients with RCP due to spinal metastases. Participants were randomly allocated to the oxycodone alone group (Group A, *n* = 30) or the oxycodone combined with PRF group (Group B, *n* = 30). Primary outcomes comprised pain scores on the Numerical Rating Scale (NRS) and episodes of breakthrough pain at post-treatment time points Secondary endpoints included opioid consumption, immune parameters, quality of life, and adverse event incidence.

**Results:**

Group B had significantly lower NRS scores and fewer 24-h breakthrough pain episodes than Group A at post-treatment time points (*p* < 0.05). The opioid consumption and 7/30-day drug escalation indices of Group B were significantly lower (*p* < 0.05), with a lower dose escalation rate (46.67% vs. 73.33%, *p* = 0.035). Group B exhibited relatively better preserved selected T lymphocyte subset parameters (CD3⁺, CD4⁺ T cell percentages, CD4⁺/CD8⁺ ratio) and quality of life scores (*p* < 0.05), with a markedly lower overall adverse reaction rate (43.33% vs. 70.00%, *p* = 0.037) and no serious adverse events observed.

**Conclusions:**

This exploratory pilot randomized controlled trial indicated that the combination of oxycodone and PRF may contribute to improved pain control, decreased opioid consumption, and attenuated decline in lymphocyte subsets alongside enhanced quality of life in patients with spinal metastasis-related refractory cancer pain. No serious adverse events were documented. The clinical and oncological significance of the observed intergroup differences in T cell indices remains unclear. These preliminary observations require verification in larger prospective trials.

**Supplementary Information:**

The online version contains supplementary material available at https://doi.org/10.1007/s00520-026-11122-x.

## Introduction

Cancer pain is one of the most prevalent clinical symptoms in patients with advanced malignant tumors, which seriously impairs their quality of life (QOL) [1, 2]. Bone metastasis represents a leading cause of chronic cancer pain [3]. The spine is the most common site of bone metastasis, accounting for approximately 70% of all bone metastases, and it often leads to severe and refractory pain [4]. Although the World Health Organization (WHO) three-step analgesic ladder and the National Comprehensive Cancer Network (NCCN) guidelines offer an effective framework for cancer pain management, there are still 10–20% of patients who experience refractory cancer pain (RCP) despite optimal pharmacotherapeutic intervention [5–7]. Pharmacological intervention based on opioids is presently the treatment of choice for cancer pain [8]. Oxycodone’s reputation as a strong opioid with high efficacy and tolerability has made its wide use commonplace [9]. Dose-limiting adverse effects, namely constipation, nausea, and somnolence often limit the achievement of adequate analgesia [10].

Interventional pain management strategies serve as an important adjuvant approach to systemic analgesia for cancer-related pain [11]. Pulsed radiofrequency (PRF) is a minimally invasive neuromodulation technique that delivers radiofrequency energy in short bursts, and does not induce thermal nerve injury [12]. Its analgesic mechanism is thought to involve the modulation of neuronal excitability via electromagnetic fields on neuronal membranes, the alteration of synaptic transmission processes, and the inhibition of pathological pain signal conduction within neural pathways [13]. Clinical evidence has confirmed that PRF exerts definite therapeutic effects on a range of neuropathic pain conditions, such as trigeminal neuralgia, postherpetic neuralgia, and radicular pain, and it has been proven to have a favorable safety profile in clinical application [14–16]. However, clinical evidence on the combination of PRF and opioid therapy for the specific management of refractory spinal metastatic cancer pain remains limited and requires further investigation.

The clinical rationale for combining pharmacological and interventional therapy for RCP stems from the complementary mechanisms of action the two modalities and potential synergistic analgesic effects. Opioid medications exert analgesic effects primarily by acting on central opioid receptors to modulate central pain perception, while PRF exerts its therapeutic effect by targeting and modulating the peripheral pain transmission pathways. This dual analgesic strategy is anticipated to achieve superior pain control while reducing clinical opioid dosages. However, high-quality randomized controlled evidence specifically exploring combined oxycodone and PRF for spinal metastatic refractory cancer pain remains scarce and incomplete. For this reason, we carried out this exploratory pilot randomized controlled trial to fill this research gap. The primary objective was to compare the analgesic efficacy of oxycodone combined with PRF versus oxycodone monotherapy in patients with refractory cancer pain due to spinal metastases. The secondary objectives were to assess between-group differences in opioid consumption, health-related quality of life, functional status, psychological state, and safety profiles of the two treatment regimens.

## Materials and methods

### Research design and participants

This randomized controlled trial was conducted at The Affiliated Yangming Hospital of Ningbo University (Yuyao People's Hospital) from October 2024 to December 2025. This study was approved by the institutional ethics committee of the hospital (Approval No. 2023-12-006) and registered in the Chinese Clinical Trial Registry (http://www.chictr.org.cn/) on 2024-09-12 (Registration No. ChiCTR2400089641). All patients signed a written informed consent form before being enrolled. RCP was diagnosed according to the Expert Consensus on Refractory Cancer Pain (2017 Edition) [17]: (1) a continuous pain Numerical Rating Scale (NRS) score ≥ 4 and/or ≥ 3 episodes of breakthrough pain per day; (2) following the WHO three-step analgesic ladder and NCCN Adult Cancer Pain Guidelines, after 1–2 weeks of standardized treatment with opioids alone or in combination with adjuvant analgesics, pain relief remains unsatisfactory or intolerable drug-related adverse reactions occur. To confirm the diagnosis of refractory cancer pain according to the Chinese expert consensus, all participants received standardized oxycodone titration before enrolment, targeting an NRS score ≤ 3. Adjuvant analgesics (gabapentinoids or antidepressants) were optimized for neuropathic-related pain components. Radiotherapy and bone-modifying agents were applied following standard clinical guidelines whenever clinically indicated. Patients were enrolled only when pain relief remained unsatisfactory despite optimized analgesic regimens, or when patients developed intolerable opioid-related adverse effects that prevented further dose escalation.

### Inclusion and exclusion criteria

Inclusion criteria are as follows: (1) pathologically diagnosed with spinal metastatic tumor; (2) aged 18 to 75 years, no gender restrictions; (3) the cause of the pain was clearly identified as spinal metastatic tumor, with the main symptom being somatic pain; (4) the vertebral lesion was not completely destroyed, and the Frankel spinal cord injury grade was D or E; (5) no severe abnormalities in blood clotting function; (6) cardiac function classified as NYHA grade ≤ 3, and the patient could tolerate the supine position for ≥ 2 h; (7) expected survival time ≥ 3 months. All participants completed standardized pharmacological analgesic trials before enrolment according to consensus-based requirements. Detailed analgesic titration procedures are described above.

Exclusion criteria are as follows: (1) individuals with cognitive impairments, mental disorders, or consciousness disorders due to intracranial metastasis of tumors; (2) those with a history of alcohol abuse, drug abuse, or opioid abuse; (3) those with a history of allergic diseases or allergic constitution, or hypersensitivity to the study-related drug components; (4) those with an infection at the pulsed radiofrequency puncture site.

### Sample size estimation

The primary endpoint of this study was the NRS score at different time points after treatment. Two-factor repeated measures analysis of variance was used, and the sample size was calculated using the G-power software (version 3.1.9.7, Heinrich Heine University Düsseldorf, Germany). The assumed effect size f was set at 0.4 (corresponding to a moderate-to-large effect based on previous pilot data), the significance level α was 0.05 (two-tailed), and the statistical power 1-β was 0.95. After calculation, 54 patients were required to be included. Considering a 10% anticipated dropout rate, we ultimately enrolled 60 patients (30 per group) to ensure adequate statistical power and allow for potential protocol deviations.

### Randomization and masking

A computerized random number generator (SPSS 26.0) produced 60 randomized codes, and the random sequence was generated by the study member responsible for statistical analysis without participating in patient recruitment or outcome assessment. Each code corresponded to either Group A (oxycodone monotherapy) or Group B (oxycodone combined with PRF therapy) treatment labels. Each code and matched treatment assignment was sealed inside sequentially numbered, opaque, tamper-proof envelopes to ensure allocation concealment. After the patient signed informed consent, the recruiting attending clinician opened envelopes consecutively according to enrollment sequence to confirm the assigned regimen before initiating standardized treatment. This study employed a single-blind design: Given the invasive nature of pulsed radiofrequency, treating operators and participants could not be blinded to group allocation. By contrast, dedicated follow-up outcome assessors and statistical analysts remained fully blinded to group assignments until data collection and statistical analyses were completed.

### Intervention plan

Both groups of patients received conventional anti-tumor treatments from the oncology department and additionally underwent pain relief treatment.

Group A: Morphine immediate-release tablets were administered for pain titration, with a target NRS score of ≤ 3. The effective analgesic dose after titration was recorded. The morphine dose was then converted to the equivalent dose of oxycodone hydrochloride sustained-release tablets (OxyContin) for oral administration every 12 h. During the treatment period, morphine immediate-release tablets were administered for rescue treatment if breakthrough pain occurred.

Group B: Based on the oxycodone treatment plan of Group A, plus ultrasound-guided pulsed radiofrequency (PRF) targeting the symptomatic spinal nerve root. Prior to PRF intervention, diagnostic nerve block was performed to identify the dominant symptomatic spinal nerve. Ultrasound guidance was performed using a Sonosite M-Turbo platform (Sonosite, USA). For cervical segments, patients were positioned supine with the head turned to the unaffected side, scanned via a high-frequency linear probe (6–13 MHz); a low-frequency curvilinear probe (2–5 MHz) was adopted for prone-positioned thoracic and lumbar patients. In very rare circumstances, bilateral treatment at the identical vertebral level or treatment covering two contiguous vertebral segments was permitted, though nearly all enrolled patients received intervention targeting a single unilateral symptomatic spinal nerve.

Peripheral venous access was established pre‑operatively. All patients received standard intraoperative monitoring including electrocardiography, pulse‑oxygen saturation, and non‑invasive blood pressure in the procedure room. After routine skin disinfection and draping, local subcutaneous infiltration anesthesia with 3 mL of 2% lidocaine was administered. PRF (An inomed Medizintechnik 22G × 100 × 5 mm RF cannula was used for puncture.) was performed under real-time ultrasound guidance according to the involved spinal segments (cervical, thoracic or lumbar). Standard PRF technical parameters were set as follows: the radiofrequency thermal coagulator was Beijing Beiqi Company, model R-2000B A1, frequency 420 kHz, voltage 45 V, pulse width 20 ms, intermission 480 ms, target temperature 42 °C, 120 s per cycle, two cycles for each target level. Detailed puncture procedures, cannula‑related information and representative ultrasound images are provided in Supplementary Materials (Figure S1‑S6). After the procedure, the cannula was removed, sterile dressing was applied, and all patients were monitored for 2 h to observe immediate-onset complications such as bleeding and neurological deficits.

### Concomitant pain management

Concomitant pain management and supportive care were standardized across both groups according to institutional protocols. All patients continued to receive their baseline antitumor therapies, including chemotherapy, radiotherapy, or targeted therapy, as determined by their oncologists, and the treatment regimens remained unchanged during the 30-day follow-up period unless clinically indicated otherwise. For breakthrough pain, morphine immediate-release tablets (5–10 mg per dose) were administered as rescue medication, with a maximum of four doses per day; the total rescue dose was recorded and included in the daily MED calculation. Adjuvant analgesics, including non-steroidal anti-inflammatory drugs (NSAIDs), gabapentinoids, or corticosteroids, were not routinely prescribed but could be added at the discretion of the treating physician for specific indications (e.g., inflammatory pain or neuropathic components); any use of these agents was documented and analyzed as a potential confounder. Baseline bone-modifying agents were maintained; no new ones were started during the 30-day follow-up. Patients requiring radiotherapy or radioactive seed implantation to the target vertebral lesions during the follow-up period were excluded from the final analysis to avoid confounding the pain outcomes. All concomitant medications and interventions were prospectively recorded in case report forms and reviewed by the independent safety monitor.

### Observation indicators and follow-up

Primary outcomes are as follows: Pain intensity was assessed using the Numerical Rating Scale (NRS, ranging from 0 = no pain to 10 = worst imaginable pain)[18]. Assessments were performed at baseline and at 1 h, 24 h, 72 h, 7 days, and 30 days post-treatment. Both resting pain and breakthrough pain episodes were recorded. Breakthrough pain was defined as transient exacerbations of pain occurring on a background of controlled baseline pain.

Secondary outcomes are as follows: (1) Opioid consumption and dose escalation index. Daily oxycodone intake was recorded and subsequently converted into oral morphine equivalent dose (MED) using standard conversion (1 mg oxycodone = 1.5 mg oral morphine). To evaluate changes in opioid requirement over time, the opioid escalation index (OEI) at day 7 and day 30 was calculated according to the following formula: OEI (%) = [(daily MED at the target time point−daily MED at baseline)/daily MED at baseline]/number of days × 100. In addition, the proportion of patients showing any increase in MED from baseline was recorded as requiring dose escalation. (2) Immune function. Peripheral venous blood samples were obtained at baseline, day 7, and day 30. T lymphocyte subsets, including the percentages of CD3⁺, CD4⁺, and CD8⁺ cells as well as the CD4⁺/CD8⁺ ratio, were measured by flow cytometry. (3) Quality of life. Health-related quality of life was assessed at baseline and on day 30 using the European Organization for Research and Treatment of Cancer Quality of Life Questionnaire-Core 30 (EORTC QLQ-C30, version 3.0) [19]. Five functional domains (physical, role, emotional, cognitive, and social functioning) were evaluated; higher scores indicate better function. (4) Safety and adverse events. All adverse events occurring during the 30-day follow-up were documented and categorized. We recorded both common opioid-related side effects (constipation, nausea/vomiting, decreased appetite, dizziness, somnolence, pruritus, and urinary retention) and procedure-related events (puncture site pain, infection, bleeding, and hematoma). All events received appropriate symptomatic management; their severity and outcomes were also noted.

### Statistical analysis

Data analysis was conducted using SPSS 26.0 statistical software (IBM Corp., Armonk, NY, USA). Continuous variables were expressed as mean ± standard deviation (SD) or median (interquartile range) depending on the distribution. Categorical variables were expressed as frequency (percentage). Between-group comparisons of continuous variables were performed using the independent samples *t*-test or Mann-Whitney *U* test. Categorical variables were compared using the Chi-square test or Fisher’s exact test. Repeated measures analysis of variance (ANOVA) was used to analyze the dynamic changes in continuous indicators (pain scores, breakthrough pain episodes, opioid consumption, immune function indices) at multiple time points, with Mauchly’s test for sphericity; the Greenhouse-Geisser correction was applied if sphericity was violated. Given the exploratory nature of the secondary outcomes and the limited sample size, no adjustments for multiple comparisons were applied. All secondary analyses should be considered hypothesis-generating, and the results should be interpreted with caution. A two-tailed *p* < 0.05 was considered statistically significant for all tests.

## Results

### Patient characteristics

**Fig. 1:**
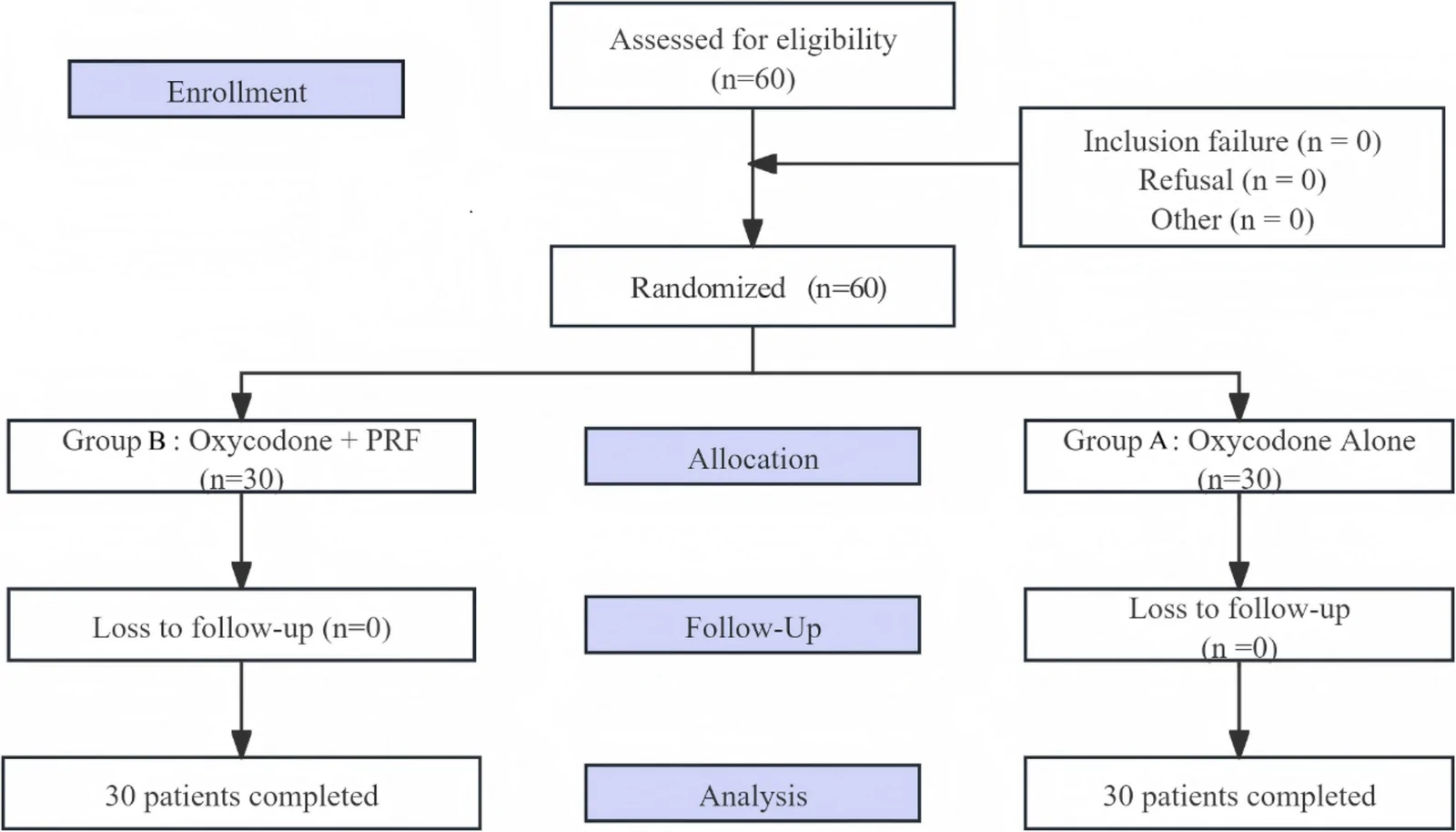
CONSORT 2010 flow diagram of the randomized controlled trial. Abbreviations: PRF, pulsed radiofrequency

The Consolidated Standards of Reporting Trials (CONSORT) flow diagram for this study is depicted in Fig. 1. A total of 60 patients with refractory cancer pain secondary to spinal metastases were enrolled and randomly assigned to either the oxycodone alone group (Group A, *n* = 30) or the oxycodone combined with pulsed radiofrequency group (Group B, *n* = 30). The baseline demographic and clinical characteristics of the patients are summarized in Table 1. The mean age of the study population was 61.42 ± 7.91 years, with 53.33% being female. No significant differences were observed between the two groups in terms of age, BMI, baseline breakthrough pain frequency, baseline NRS score, gender distribution, Frankel grade, prior radiotherapy, prior chemotherapy, or number of metastatic sites (all *p* > 0.05), indicating that the two groups were well-balanced at baseline.

**Table 1:** Baseline characteristics of the study population

Variables	Total (*n* = 60)	Group A (*n* = 30)	Group B (*n* = 30)	Statistic	*p*
Age (years), Mean ± SD	61.42 ± 7.91	62.03 ± 7.61	60.80 ± 8.29	*t* = 0.60	0.551
BMI (kg/m^2^), Mean ± SD	23.20 ± 3.22	23.81 ± 3.26	22.58 ± 3.11	*t* = 1.49	0.141
Baseline breakthrough pain (episodes/day), Mean ± SD	4.75 ± 2.14	4.67 ± 2.06	4.83 ± 2.26	*t* = −0.30	0.766
Baseline NRS, Mean ± SD	6.80 ± 0.51	6.77 ± 0.57	6.83 ± 0.46	*t* = −0.50	0.620
Gender, *n*(%)				*χ*^*2*^ = 1.07	0.301
Female	32 (53.33)	14 (46.67)	18 (60.00)		
Male	28 (46.67)	16 (53.33)	12 (40.00)		
Frankel grade, *n*(%)				*χ*^*2*^ = 0.69	0.405
D	19 (31.67)	11 (36.67)	8 (26.67)		
E	41 (68.33)	19 (63.33)	22 (73.33)		
Prior radiotherapy, *n*(%)				*χ*^*2*^ = 0.08	0.781
No	19 (31.67)	10 (33.33)	9 (30.00)		
Yes	41 (68.33)	20 (66.67)	21 (70.00)		
Prior chemotherapy, *n*(%)				*χ*^*2*^ = 0.00	1.000
No	14 (23.33)	7 (23.33)	7 (23.33)		
Yes	46 (76.67)	23 (76.67)	23 (76.67)		
Metastatic sites, *n*(%)				*χ*^*2*^ = 4.330	0.215^a^
Thoracic spine	23 (38.33)	8 (26.67)	15 (50.00)		
Cervical spine	16 (26.67)	10 (33.33)	6 (20.00)		
Lumbar spine	16 (26.67)	10 (33.33)	6 (20.00)		
Multiple segments	5 (8.33)	2 (13.33)	3 (10.00)		

^a^Fisher’s exact test

*BMI* body mass index, *NRS* numerical rating scale, *SD* standard deviation

### Temporal changes in pain intensity

The longitudinal changes in pain intensity (NRS scores) over the 30-day follow-up period are presented in Table 2. At baseline, the mean NRS scores were comparable between Group A (6.77 ± 0.57) and Group B (6.83 ± 0.46) (p = 0.620). Following treatment, both groups showed progressive reductions in pain scores over time. Although no significant difference was observed at 1 h or 24 h post-treatment, Group B demonstrated significantly lower pain scores compared to Group A starting from 72 h (2.47 ± 0.68 vs. 2.93 ± 0.64, *p* = 0.008), and this difference persisted at 7 days (1.83 ± 0.59 vs. 2.37 ± 0.67, *p* = 0.002) and 30 days (1.57 ± 0.50 vs. 2.07 ± 0.45, *p* < 0.001). Repeated measures ANOVA revealed significant main effects for group (*F* = 33.61, *p* < 0.001) and time (*F* = 533.76, *p* < 0.001) and a significant group-by-time interaction (*F* = 2.28, *p* = 0.047), indicating pain reduction trajectory was significantly different between both groups over the study period.

**Table 2:** Longitudinal changes in pain intensity (NRS scores)

Variables	Total (*n* = 60)	Group A (*n* = 30)	Group B (*n* = 30)	Statistic	*p*
Baseline, Mean ± SD	6.80 ± 0.51	6.77 ± 0.57	6.83 ± 0.46	*t* = −0.50	0.620
1 h, Mean ± SD	4.23 ± 0.67	4.30 ± 0.70	4.17 ± 0.65	*t* = 0.76	0.448
24 h, Mean ± SD	3.75 ± 0.60	3.87 ± 0.68	3.63 ± 0.49	*t* = 1.52	0.133
72 h, Mean ± SD	2.70 ± 0.70	2.93 ± 0.64	2.47 ± 0.68	*t* = 2.73	0.008
7 days, Mean ± SD	2.10 ± 0.68	2.37 ± 0.67	1.83 ± 0.59	*t* = 3.27	0.002
30 days, Mean ± SD	1.82 ± 0.54	2.07 ± 0.45	1.57 ± 0.50	*t* = 4.05	< 0.001
*F* value	*F*-group = 33.64, *F*-time = 533.76, *F*-interaction = 2.28				
*p* value	*p*-group < 0.001, *p*-time < 0.001, *p*-interaction = 0.047				

Mauchly *W* = 0.73, *p* = 0.238

*NRS* numerical rating scale, *SD *standard deviation

### Changes in breakthrough pain episodes

Table 3 summarizes the frequency of breakthrough pain episodes over time. At baseline, the mean daily breakthrough pain episodes were similar between Group A (4.67 ± 2.06) and Group B (4.83 ± 2.26) (*p* = 0.766). Group B exhibited significantly fewer breakthrough pain episodes compared to Group A at day 3 (1.30 ± 0.65 vs. 1.63 ± 0.56, *p* = 0.037), day 7 (0.73 ± 0.52 vs. 1.07 ± 0.58, *p* = 0.023), and day 30 (0.77 ± 0.43 vs. 1.10 ± 0.48, *p* = 0.006). Findings from the repeated measures ANOVA revealed a significant main effect of time (*F* = 69.16, *p* < 0.001), suggesting that breakthrough pain frequency decreased significantly over the follow-up in both groups. However, the main effect of the group was not statistically significant (*F* = 3.68, *p* = 0.060), and there was no significant group-by-time interaction (*F* = 0.18, *p* = 0.947).

**Table 3:** Longitudinal changes in breakthrough pain episodes

Variables	Total (*n* = 60)	Group A (*n* = 30)	Group B (*n* = 30)	Statistic	*p*
Baseline, Mean ± SD	4.75 ± 2.14	4.67 ± 2.06	4.83 ± 2.26	*t* = −0.30	0.766
Day 1, Mean ± SD	2.23 ± 1.01	2.40 ± 1.16	2.07 ± 0.83	*t* = 1.28	0.206
Day 3, Mean ± SD	1.47 ± 0.62	1.63 ± 0.56	1.30 ± 0.65	*t* = 2.13	0.037
Day 7, Mean ± SD	0.90 ± 0.57	1.07 ± 0.58	0.73 ± 0.52	*t* = 2.33	0.023
Day 30, Mean ± SD	0.93 ± 0.48	1.10 ± 0.48	0.77 ± 0.43	*t* = 2.83	0.006
*F* value	*F*-group = 3.68, *F*-time = 69.16, *F*-interaction = 0.18				
*p* value	*p*-group = 0.060, *p*-time < 0.001, *p*-interaction = 0.947				

Mauchly *W* = 0.02, *p* < 0.001

*SD* standard deviation

**Fig. 2:**
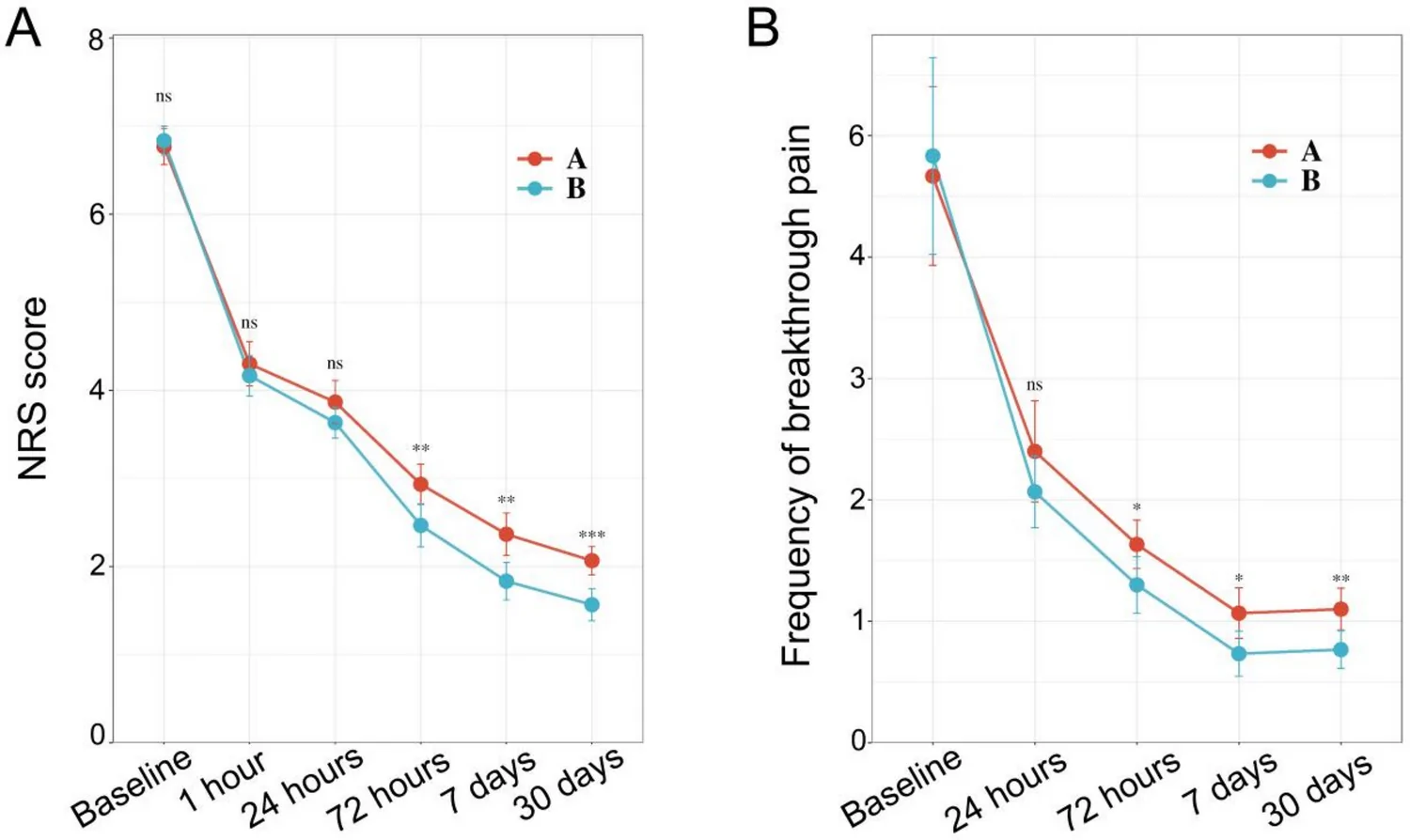
Longitudinal changes in pain indicators at baseline and post-treatment. **A** NRS score. **B** Frequency of breakthrough pain. ns, not significant; *p < 0.05; **p < 0.01; ***p < 0.001

To better visualize the dynamic changes in pain control outcomes, the longitudinal trends in NRS scores and breakthrough pain episodes are illustrated in Fig. 2.

### Opioid consumption and dose escalation index

Table 4 summarizes opioid use (as daily OME) and the OEI at days 7 and 30. No difference was seen at baseline between Group A (83.15 ± 26.31 mg) and Group B (85.74 ± 23.91 mg) (*p* = 0.692). By day 7, however, patients receiving combination therapy required significantly less opioid than those on oxycodone alone (92.48 ± 23.16 mg vs. 107.52 ± 27.71 mg, *p* = 0.026), and this gap widened further by day 30 (96.18 ± 28.34 mg vs. 125.37 ± 36.52 mg, *p* = 0.001). The OEI was also markedly lower in Group B, both at day 7 (1.26 ± 1.37%/day vs. 4.71 ± 2.95%/day, *p* < 0.001) and at day 30 (0.40 ± 0.28%/day vs. 1.81 ± 0.71%/day, *p* < 0.001). Consistently, the proportion of patients who required any dose escalation was smaller in Group B (46.67% vs. 73.33%, *p* = 0.035). Repeated-measures ANOVA confirmed a strong time effect (*F* = 305.34, *p* < 0.001) and, more importantly a significant group-by-time interaction (*F* = 104.12, *p* < 0.001), indicating that the two groups followed distinctly different trajectories of opioid consumption over the 30-day study period.

**Table 4:** Opioid consumption and dose escalation index

Variables	Total (*n* = 60)	Group A (*n* = 30)	Group B (*n* = 30)	Statistic	*p*
Baseline daily MED (mg), Mean ± SD	84.44 ± 24.96	83.15 ± 26.31	85.74 ± 23.91	*t* = −0.40	0.692
Day 7 daily MED (mg), Mean ± SD	100.00 ± 26.43	107.52 ± 27.71	92.48 ± 23.16	*t* = 2.28	0.026
Day 30 daily MED (mg), Mean ± SD	110.78 ± 35.59	125.37 ± 36.52	96.18 ± 28.34	*t* = 3.46	0.001
Day 7 OEI (%/day), Mean ± SD	2.98 ± 2.87	4.71 ± 2.95	1.26 ± 1.37	*t* = 5.81	< 0.001
Day 30 OEI (%/day), Mean ± SD	1.10 ± 0.89	1.81 ± 0.71	0.40 ± 0.28	*t* = 10.22	< 0.001
Patients requiring dose escalation	36 (60.00)	22 (73.33)	14 (46.67)	*χ*^*2*^ = 4.44	0.035
*F* value	*F*-group = 3.83, *F*-time = 305.34, *F*-interaction = 104.12				
*p* value	*p*-group = 0.055, *p*-time < 0.001, *p*-interaction < 0.001				

Mauchly *W* = 0.646, *p* < 0.001

*MED* morphine equivalent dose, *OEI* opioid escalation index, *SD* standard deviation

### Dynamic changes in T lymphocyte subset parameters

The changes in immunological parameters over time are summarized in Table 5. At baseline, no significant differences were observed between the two groups in any of the immunological markers, including CD3⁺, CD4⁺, CD8⁺ T cell percentages, and the CD4⁺/CD8⁺ ratio (all *p* > 0.05). At day 7, Group B demonstrated significantly higher percentages of CD3⁺ (55.61% ± 5.08% vs. 50.74% ± 4.82%, *p* < 0.001) and CD4⁺ cells (32.21% ± 3.42% vs. 28.37% ± 2.84%, *p* < 0.001) compared to Group A, as well as a higher CD4⁺/CD8⁺ ratio (1.26 ± 0.18 vs. 1.14 ± 0.15, *p* = 0.008). These differences persisted at day 30, with Group B showing superior CD3⁺ (60.42% ± 4.88% vs. 57.19% ± 5.13%, *p* = 0.015), CD4⁺ (35.18% ± 3.25% vs. 33.41% ± 3.17%, *p* = 0.038), and CD4⁺/CD8⁺ ratio (1.52 ± 0.24 vs. 1.39 ± 0.19, *p* = 0.026). No significant differences were observed in CD8⁺ cell percentages at any time point (all *p* > 0.05). Repeated measures ANOVA confirmed significant group effects for CD3⁺ (*p* = 0.002), CD4⁺ (*p* = 0.001), and CD4⁺/CD8⁺ ratio (*p* = 0.035), as well as significant group-by-time interactions for these parameters (all *p* < 0.05), indicating intergroup differences in the longitudinal evolution of these measured T lymphocyte subset parameters.

**Table 5:** Dynamic changes in immune function indices

Variables	Time	Total (*n* = 60)	Group A (*n* = 30)	Group B (*n* = 30)	Statistic	*p*
CD3^+^ (%)	Baseline	62.21 ± 5.39	62.53 ± 5.64	61.89 ± 5.21	*t* = 0.46	0.650
	Day 7	53.18 ± 5.49	50.74 ± 4.82	55.61 ± 5.08	*t* = −3.81	< 0.001
	Day 30	58.81 ± 5.23	57.19 ± 5.13	60.42 ± 4.88	*t* = −2.50	0.015
	*F* value	*F*-group = 10.73, *F*-time = 48.90, *F*-interaction = 4.31				
	*p* value	*p*-group = 0.002, *p*-time < 0.001, *p*-interaction = 0.018				
CD4^+^ (%)	Baseline	37.47 ± 3.43	37.76 ± 3.48	37.18 ± 3.42	*t* = 0.66	0.513
	Day 7	30.29 ± 3.67	28.37 ± 2.84	32.21 ± 3.42	*t* = −4.72	< 0.001
	Day 30	34.30 ± 3.30	33.41 ± 3.17	35.18 ± 3.25	*t* = −2.13	0.038
	*F* value	*F*-group = 11.30,* F*-time = 79.76, *F*-interaction = 7.35				
	*p* value	*p*-group = 0.001, *p*-time < 0.001, *p*-interaction = 0.001				
CD8^+^ (%)	Baseline	23.36 ± 1.94	23.15 ± 2.06	23.57 ± 1.82	*t* = −0.85	0.400
	Day 7	25.55 ± 2.66	25.17 ± 2.74	25.93 ± 2.56	*t* = −1.12	0.267
	Day 30	23.80 ± 2.31	24.17 ± 2.33	23.42 ± 2.27	*t* = 1.25	0.216
	*F* value	*F*-group = 0.17, *F*-time = 13.55, *F*-interaction = 1.95				
	*p* value	*p*-group = 0.685, *p*-time < 0.001, *p*-interaction = 0.152				
CD4^+^/CD8^+^ ratio	Baseline	1.61 ± 0.20	1.64 ± 0.20	1.59 ± 0.19	*t* = 1.13	0.264
	Day 7	1.20 ± 0.18	1.14 ± 0.15	1.26 ± 0.18	*t* = −2.74	0.008
	Day 30	1.46 ± 0.22	1.39 ± 0.19	1.52 ± 0.24	*t* = −2.28	0.026
	*F* value	*F*-group = 4.66, *F*-time = 90.24, *F*-interaction = 3.92				
	*p* value	*p*-group = 0.035, *p*-time < 0.001, *p*-interaction = 0.025				

*CD* cluster of differentiation, *SD* standard deviation

### Quality of life evaluated by EORTC QLQ-C30

Quality of life was evaluated using the EORTC QLQ-C30 questionnaire, with results summarized in Table 6. Before treatment, the two groups were well matched across all functional domains—physical, role, emotional, cognitive, and social functioning—with no statistically significant differences observed (all *p* > 0.05). At day 30, both groups showed significant improvements from baseline in all functional domains (all *p* < 0.001). However, Group B demonstrated significantly greater improvements compared to Group A in emotional functioning (81.63 ± 5.22 vs. 78.20 ± 5.77, *p* = 0.019), cognitive functioning (80.83 ± 5.19 vs. 77.50 ± 5.34, *p* = 0.017), physical functioning (77.40 ± 4.87 vs. 72.27 ± 5.30, *p* < 0.001), and social functioning (76.50 ± 6.12 vs. 71.87 ± 6.33, *p* = 0.006).

**Table 6:** Quality of life assessed by EORTC QLQ-C30

Domain	Time	Total (*n* = 60)	Group A (*n* = 30)	Group B (*n* = 30)	Statistic	*p*
Emotional functioning	Baseline	61.83 ± 3.69	61.57 ± 3.33	62.10 ± 4.06	*t* = −0.56	0.580
	Day 30	79.92 ± 5.72***	78.20 ± 5.77***	81.63 ± 5.22***	*t* = −2.42	0.019
Role functioning	Baseline	58.15 ± 5.53	58.73 ± 4.89	57.57 ± 6.12	*t* = 0.82	0.418
	Day 30	76.25 ± 6.50***	74.87 ± 6.62***	77.63 ± 6.18***	*t* = −1.67	0.100
Cognitive functioning	Baseline	72.68 ± 4.41	72.27 ± 4.30	73.10 ± 4.54	*t* = −0.73	0.468
	Day 30	79.17 ± 5.48***	77.50 ± 5.34***	80.83 ± 5.19***	*t* = −2.45	0.017
Physical functioning	Baseline	55.95 ± 4.48	55.73 ± 4.28	56.17 ± 4.73	*t* = −0.37	0.711
	Day 30	74.83 ± 5.67***	72.27 ± 5.30***	77.40 ± 4.87***	*t* = −3.91	< 0.001
Social functioning	Baseline	64.63 ± 5.39	64.37 ± 5.29	64.90 ± 5.57	*t* = −0.38	0.705
	Day 30	74.18 ± 6.60***	71.87 ± 6.33***	76.50 ± 6.12***	*t* = −2.88	0.006

***p < 0.001 compared with baseline in the same group, using a paired *t*-test

*EORTC QLQ-C30* European organisation for research and treatment of cancer quality of life questionnaire-core 30, *SD* standard deviation

### Safety and adverse events

Table 7 summarizes the safety data for both treatment groups. Fewer patients in Group B experienced adverse events compared to Group A (43.3% vs. 70.0%, *p* = 0.037). No serious adverse events or treatment-related deaths occurred during the study period, and all reported events resolved with appropriate symptomatic management. When we looked at specific side effects, the numbers in Group B were generally lower: constipation (26.7% vs. 46.7%, *p* = 0.108), nausea/vomiting (16.7% vs. 36.7%, *p* = 0.080), and dizziness (13.3% vs. 36.7%, *p* = 0.037). The difference in dizziness reached statistical significance. For other events—such as decreased appetite, somnolence, pruritus, and urinary retention—rates were similar between the two groups (all *p* > 0.05). Only two patients in Group B reported mild pain at the PRF puncture site, which went away on its own without any intervention. There were no cases of infection, bleeding, or hematoma associated with the procedure.

**Table 7:** Safety and adverse events

Adverse event	Group A (*n* = 30)	Group B (*n* = 30)	Statistic	*p*
Overall adverse events	21	13	*χ*^*2*^ = 4.34	0.037
Gastrointestinal disorders				
Constipation	14	8	*χ*^*2*^ = 2.58	0.108
Nausea/vomiting	11	5	*χ*^*2*^ = 3.07	0.080
Decreased appetite	6	4	*χ*^*2*^ = 0.480	0.488
Nervous system disorders				
Dizziness	11	4	*χ*^*2*^ = 4.36	0.037
Somnolence	7	4	*χ*^*2*^ = 1.00	0.317
Pruritus	3	2	-	1.000^a^
Urinary disorders				
Urinary retention	4	2	-	0.671^a^
PRF-related adverse events				
Puncture site pain	-	2	-	-
Infection	-	0	-	-
Bleeding/hematoma	-	0	-	-

^a^Fisher’s exact test

*PRF* pulsed radiofrequency

## Discussion

To the best of our knowledge, few randomized controlled trials have investigated the combined use of pulsed radiofrequency and oxycodone for refractory cancer pain secondary to spinal metastatic tumors. Our pilot study provides preliminary evidence in this field. Compared to opioids alone, the combination provided better pain relief—an effect that emerged by 72 h and was still evident at 30-day follow-up. The benefits, however, went beyond pain scores. Patients in the combination group also had fewer breakthrough episodes, used less opioids, showed relative preservation of lymphocyte subsets, and reported higher quality of life, with fewer adverse events noted in the combination group versus the monotherapy arm within this small pilot cohort. Taken together, these results offer preliminary clinical evidence on how to manage one of the most challenging pain conditions in oncology. Nevertheless, several inherent constraints restrict the generalizability of our outcomes, as elaborated in the Limitations section below.

The superior analgesic effect achieved with combined PRF therapy warrants further mechanistic investigation. Pain induced by spinal metastases is attributed to a complex pathophysiological process, which potentially involves two aspects: nerve root pain resulting from local tumor invasion and mechanical pain secondary to spinal instability [20, 21]. Opioid drugs exert their analgesic effect mainly by activating central μ-receptors, yet they show obvious limitations in relieving neuropathic pain [22, 23]. As a novel neuromodulation technique, PRF is hypothesized to act by delivering specific-frequency electromagnetic fields to the dorsal root ganglia and affected spinal nerves [12] It may reversibly inhibit the abnormal electrical discharge of neurons, regulate the normal function of ion channels, and modulate the expression of proinflammatory cytokines in the pain pathway [24]. This process may thereby block the conduction of pathological pain signals at the level of peripheral neural pathways. Prior studies have reported favorable analgesic outcomes of PRF for neuropathic pain disorders such as trigeminal neuralgia, with sustained pain reduction within three months of treatment [25]. Preclinical work also supports the potential utility of PRF for neuropathic pain management [26, 27]. In our research, the combined therapy group began to show a significant analgesic advantage at 72 h after treatment, which is consistent with the typical time characteristic of PRF’s onset of action. Unlike radiofrequency thermocoagulation, which provides immediate pain relief, the modulatory effects of PRF may require time to accumulate fully [28]. Combined use of the two therapies may leverage complementary advantages to produce synergistic analgesia, potentially integrating central opioid-mediated analgesia and peripheral neuromodulation of pain signaling. This dual-mode analgesic framework represents a hypothetical explanation for the improved pain control observed in the combination group, rather than a confirmed causal mechanism.

The opioid sparing effect observed in this study has significant clinical implications. The combination therapy group exhibited significantly lower oxycodone consumption at 30-day compared to the monotherapy group, and a lower proportion of patients demonstrated a positive opioid dose escalation index. This result suggests that PRF intervention may lessen the patients’ dependence on opioid drugs. Reducing the dosage of opioids can directly reduce the risk of dose-related adverse reactions [29]. In this study, the incidence rates of adverse reactions such as constipation, nausea and vomiting, and dizziness in the combined treatment group showed a downward trend, which only provides preliminary tentative support for this hypothesis within our small pilot sample. Furthermore, the lower-dose-escalation index observed in the combination group may prolong the usable therapeutic window for opioids; however, this finding cannot be directly equated with delayed opioid tolerance [30]. The main reason for this might lie in the complementary and synergistic effects of the two mechanisms. PRF may modulate spinal nerve activity, potentially reducing the transmission of pathological pain impulses caused by spinal metastasis. This may compensate for the limited efficacy of oxycodone against neuropathic pain components, though we cannot confirm whether this accounts for the observed benefits given the absence of standardized pain phenotype stratification. At the same time, PRF may reduce breakthrough pain episodes and rescue-medication requirements, which could contribute to lower baseline oxycodone dosage; nevertheless, we cannot confirm that this reflects true attenuation of opioid tolerance [31].

Another notable finding of this study is divergent longitudinal changes in circulating T lymphocyte subsets between the two treatment groups. Patients with advanced tumors are already in a state of impaired immune function [32], and the administration of high-dose opioids will further inhibit the proliferation and activation of T lymphocytes in the body. This not only leads to a decrease in the levels of CD3^+^ and CD4^+^ T cells, but also disrupts the balance of the CD4^+^/CD8^+^ ratio, thereby exacerbating the systemic immune suppression [33]. In the combined therapy, the synergistic analgesic effect of PRF may markedly reduce the required dosage of opioids for patients, which in turn could alleviate the immunosuppressive side effects caused by opioid drugs. As a minimally invasive interventional technique, PRF causes only slight trauma to the body and does not trigger an obvious systemic stress response, thus avoiding adding extra physical burden to patients with advanced tumors [34] in comparison with surgical treatment or high-dose radiotherapy. The observed intergroup difference in CD4⁺/CD8⁺ ratio only reflects changes in limited peripheral T cell indices. Importantly, we cannot conclude that this alteration represents improved overall cellular immune balance, nor can we infer enhanced anti-tumor immunity or delayed tumor progression based solely on these limited laboratory measurements [35]. The above immune-related observations may carry clinical implications for optimizing quality of life among patients with terminal malignancies. In addition, the minimally invasive characteristic of PRF means that the adverse reactions associated with this procedure are mild and well-tolerated. In this study, only two patients in the combined therapy group experienced mild pain at the puncture site after PRF treatment, and no serious complications such as local infection or bleeding were observed. These findings suggest acceptable safety and tolerability of PRF among patients with advanced tumors in the present cohort.

The present work should be regarded as an exploratory pilot randomized controlled trial. This study has several limitations. Firstly, the sample size is relatively small. Although a strict sample size estimation was conducted, subgroup analysis was not performed to explore the impact of different tumor types and metastatic sites on treatment response. Furthermore, given the small sample size, our study is under‑powered to detect rare but clinically relevant adverse events associated with PRF. Secondly, the 30-day follow-up period is relatively short, which limits our ability to evaluate the sustained analgesic effect of PRF as well as long-term safety outcomes. Thirdly, pulsed radiofrequency is an invasive interventional procedure, so this study adopted a single-blind design, which cannot fully eliminate implementation bias and measurement bias for subjective endpoints including pain intensity and quality of life. Fourthly, this study did not set up a sham surgery control group, which restricts the interpretation of subjective outcomes (pain scores, breakthrough pain, quality of life) and cannot fully exclude potential placebo and patient expectation effects. Such expectation bias may partially inflate the observed analgesic benefit of PRF in our cohort. Fifthly, only T-lymphocyte subsets were measured in this study, without assessing additional immune markers such as natural killer cell activity and inflammatory cytokine profiles. Furthermore, clinically relevant endpoints including infection incidence, tumor progression and survival outcomes were not evaluated. Therefore, the clinical and oncological significance of the observed intergroup differences in T cell indices remains unclear. Sixth, validated neuropathic pain tools (DN4, PainDETECT) were not applied for prospective pain phenotype stratification. All participants completed standardized opioid titration before enrollment; nociceptive pain was largely controlled, and residual refractory pain was predominantly paroxysmal with clinical features suggestive of neuropathic pain. Without formal scale evaluation, we cannot confirm whether treatment benefits stemmed from alleviation of neuropathic pain components. Standardized pain phenotyping will be implemented in future trials. Seventhly, as an exploratory pilot trial, no multiple-comparison correction was performed for multiple secondary endpoints, which may lead to inflation of type-I error. Therefore, findings from secondary outcomes need further validation in future formal clinical trials. Future research should focus on the following aspects: conduct multicenter, large-sample randomized controlled trials, and perform subgroup analysis based on tumor types and metastatic sites; extend the follow-up period to more than 6 months to evaluate the persistence of efficacy; adopt double-blind, sham surgery control design to further confirm the efficacy; include more comprehensive immunological and pain biomarker detection to explore the predictive factors of PRF efficacy; conduct health economics evaluation to provide a basis for clinical decision-making and medical insurance policies. In addition, research on the optimization of different PRF parameters (voltage, frequency, treatment time) will also help to further improve the treatment effect.

## Conclusion

In conclusion, this exploratory pilot trial preliminarily suggests that the combination of oxycodone and pulsed radiofrequency therapy for RCP caused by spinal metastases may facilitate pain relief, lower opioid consumption, attenuate the decline in lymphocyte subsets, and improve quality of life, without obvious safety concerns. These preliminary observations may offer modest insights for refractory cancer pain management. Larger multicenter, ideally double-blind, sham-controlled trials are warranted to validate our findings prior to recommending widespread clinical implementation.

## Supplementary Information

Below is the link to the electronic supplementary material.

ESM 1 (PDF 407 KB)

## Data Availability

The datasets used and/or analysed during the current study are available from the corresponding author on reasonable request.

## Declarations

### Clinical trial registration

Chinese Clinical Trial Registry (http://www.chictr.org.cn/), Registration date: 2024-09-12, Registration No.: ChiCTR2400089641
