# Supplementary material for: Oxycodone combined with pulsed radiofrequency for refractory cancer pain from spinal metastases: a randomized controlled study
Source: Support Care Cancer. 2026 Aug 24;34(9):896. doi: 10.1007/s00520-026-11122-x (PMC13503447; doi:10.1007/s00520-026-11122-x)
Supplement: Supplementary file 1 [file 520_2026_11122_MOESM1_ESM.pdf]

Supplementary Material:

Standardized Ultrasound-Guided Pulsed Radiofrequency (PRF) for Spinal Nerve Root

Prior to PRF intervention, diagnostic dermatomal nerve blocks were performed to identify the single dominant symptomatic spinal nerve root corresponding to each patient's metastatic spinal lesion. All procedures were completed under real-time ultrasound guidance with full sterile disinfection and draping protocols. Patient positioning was individualized by target spinal segment: patients with cervical lesions were placed supine with the head rotated toward the unaffected side; thoracic and lumbar patients were positioned prone with thin supportive padding under the chest or abdomen to optimize paravertebral ultrasound visualization.

After full sterile skin disinfection and draping, 3 mL of 2% lidocaine was injected for subcutaneous infiltration anesthesia along the planned puncture track. All ultrasound guidance was implemented on a Sonosite M-Turbo platform (Sonosite, USA) with two dedicated transducers: a high-frequency linear probe (6–13 MHz) for cervical segments and a low-frequency curvilinear probe (2–5 MHz) for thoracic and lumbar segments. An inomed Medizintechnik 22G × 100 × 5 mm RF cannula was advanced under continuous real-time sonographic visualization toward the target spinal nerve root.

Segment-specific scanning protocols were implemented as follows:

1. Cervical segments: Transverse plane scanning to visualize transverse processes and intervertebral foramina.

2. Thoracic segments: Short-axis paravertebral scanning with cautious needle advancement to minimize pleural injury risk.

3. Lumbar segments: Sequential identification of spinous and transverse processes to locate target intervertebral foramina for target spinal nerve root access.

Once satisfactory cannula positioning adjacent to the target spinal nerve root was

confirmed, standardized PRF therapy was performed using a Beijing Beiqi R-2000B A1 radiofrequency thermal coagulator. Fixed treatment parameters were set at 420 kHz frequency, 45 V voltage, 20 ms pulse width, 480 ms intermission, and a target temperature of 42 °C; each target segment received two cycles lasting 120 s per cycle.

Nearly all participants received unilateral single-level spinal nerve root treatment; bilateral puncture or intervention across two contiguous vertebral segments was permitted only in rare clinical cases.

After treatment completion, the RF cannula was slowly withdrawn and a sterile compressive dressing placed over the puncture site. All patients were monitored for 2 hours post-procedure to screen for acute complications including hemorrhage and focal neurological deficits.

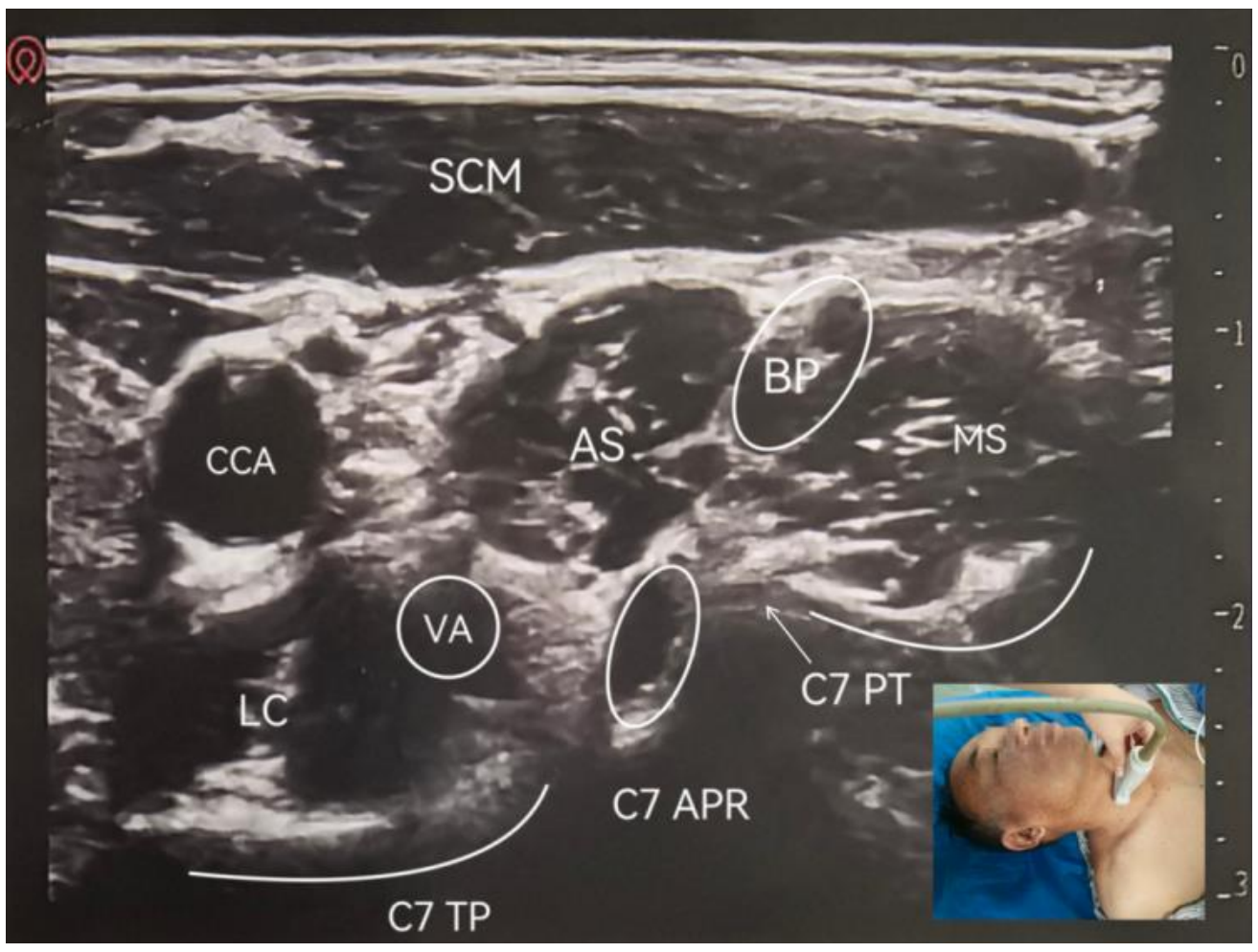

Figure S1. Ultrasound anatomical localization of the cervical spinal nerve root.

SCM = Sternocleidomastoideus muscle; CCA = Common carotid artery; LC = Longus colli muscle; C7 TP = 7th cervical vertebra Transverse process; VA = Vertebral artery; C7 APR= 7th cervical vertebra Anterior primary ramus; C7 PT = 7th cervical vertebra Posterior tubercle of transverse process; MS = Middle scalene muscle; BP= Brachial plexus; AS = Anterior scalene muscle.

Localization procedure: Patients with cervical lesions were placed in the supine position with the head turned to the healthy side. A linear ultrasound probe (6-13

MHz, Sonosite M-Turbo) was placed transversely at the cricoid cartilage level (C6 plane) and slid caudally until the anterior and posterior tubercles of the transverse process disappeared. The "beach chair"-shaped hyperechoic structure indicates C7 PT. The probe was then slid cranially until C7 TP disappeared, corresponding to the C6-7 intervertebral foramen (target level for C7 spinal nerve localization). For C3-C6 spinal nerves, the probe was slid cranially sequentially to identify corresponding transverse processes and intervertebral foramina.

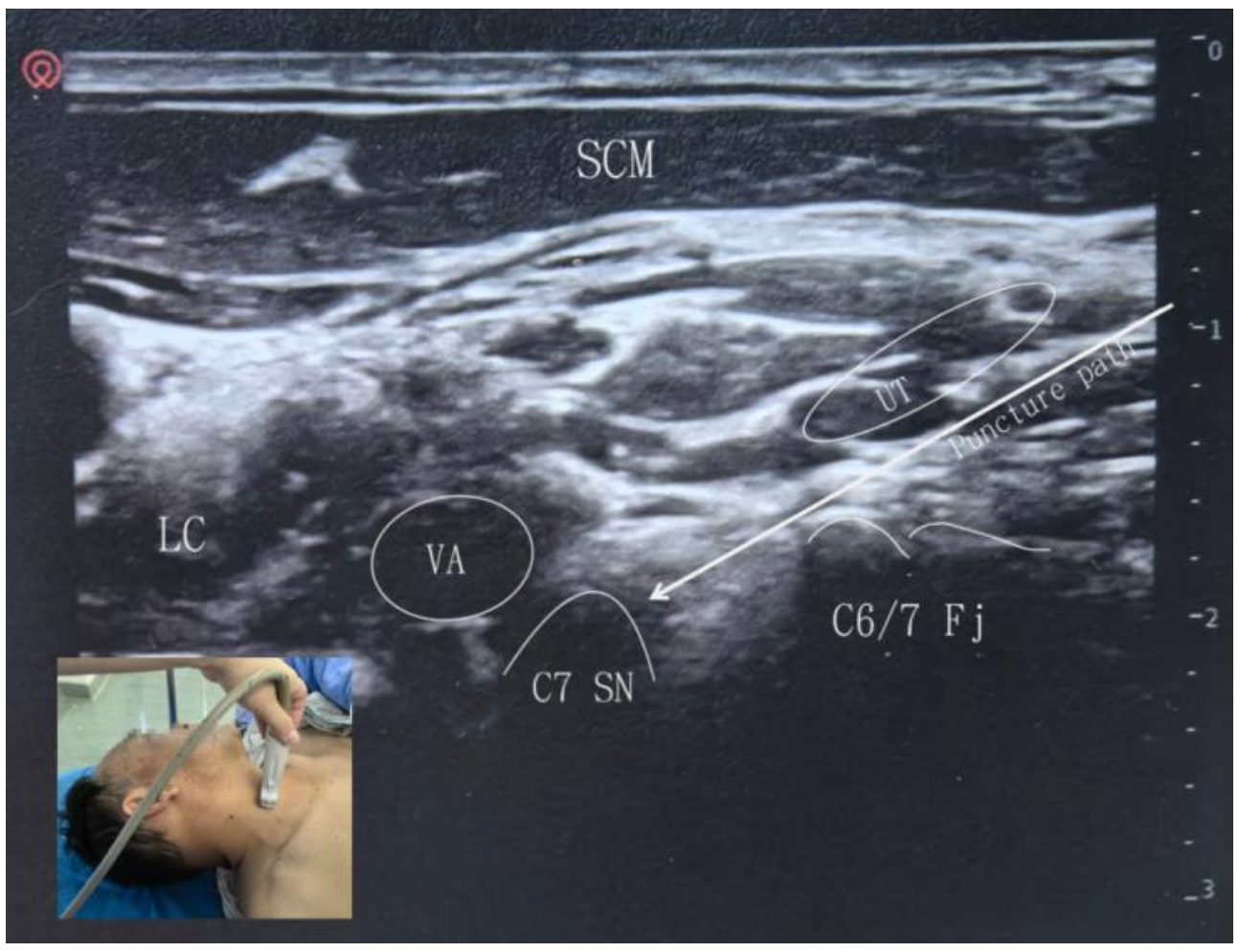

Figure S2. Ultrasound view and puncture trajectory of the C7 spinal nerve root.

LC = Longus colli muscle; VA = Vertebral artery; C7 SN = 7th cervical vertebra spinal nerve; C6/7 Fj = 6th/7th Cervical Vertebra facet joint; UT = Upper trunk of the brachial plexus;

Puncture procedure: Under ultrasound guidance, the RF cannula was advanced to the medial aspect of C6/7 Fj using the short-axis plane technique with a strictly medial needle trajectory to minimize vertebral artery and neural vascular risk.

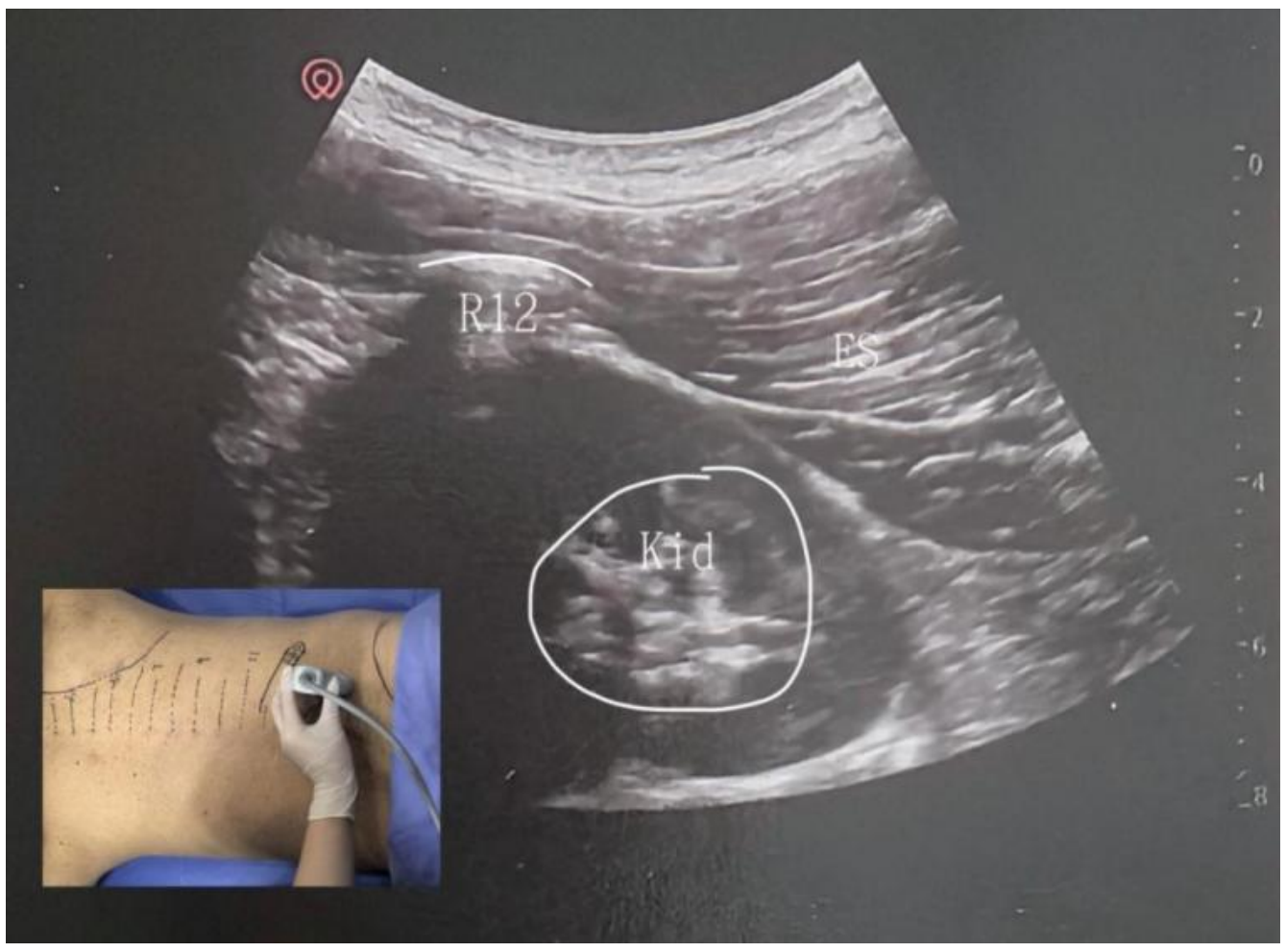

Figure S3. Landmark ultrasound scanning for thoracic spinal nerve root targeting.

R12 = Ribs 12; Kid = Kidney; ES = Erector spinae

Localization procedure: Patients were placed in the prone position with a thin pillow under the chest. A convex ultrasound probe (2-5 MHz, Sonosite M-Turbo) was placed longitudinally at the lumbar transverse process level, slid laterally until the transverse process disappeared, then cranially until the hyperechoic bony cortex (12th rib short-axis view) appeared. The probe was slid medially to identify T12 transverse process, then cranially sequentially to confirm target thoracic vertebral segments (T1-T12) using transverse processes as landmarks.

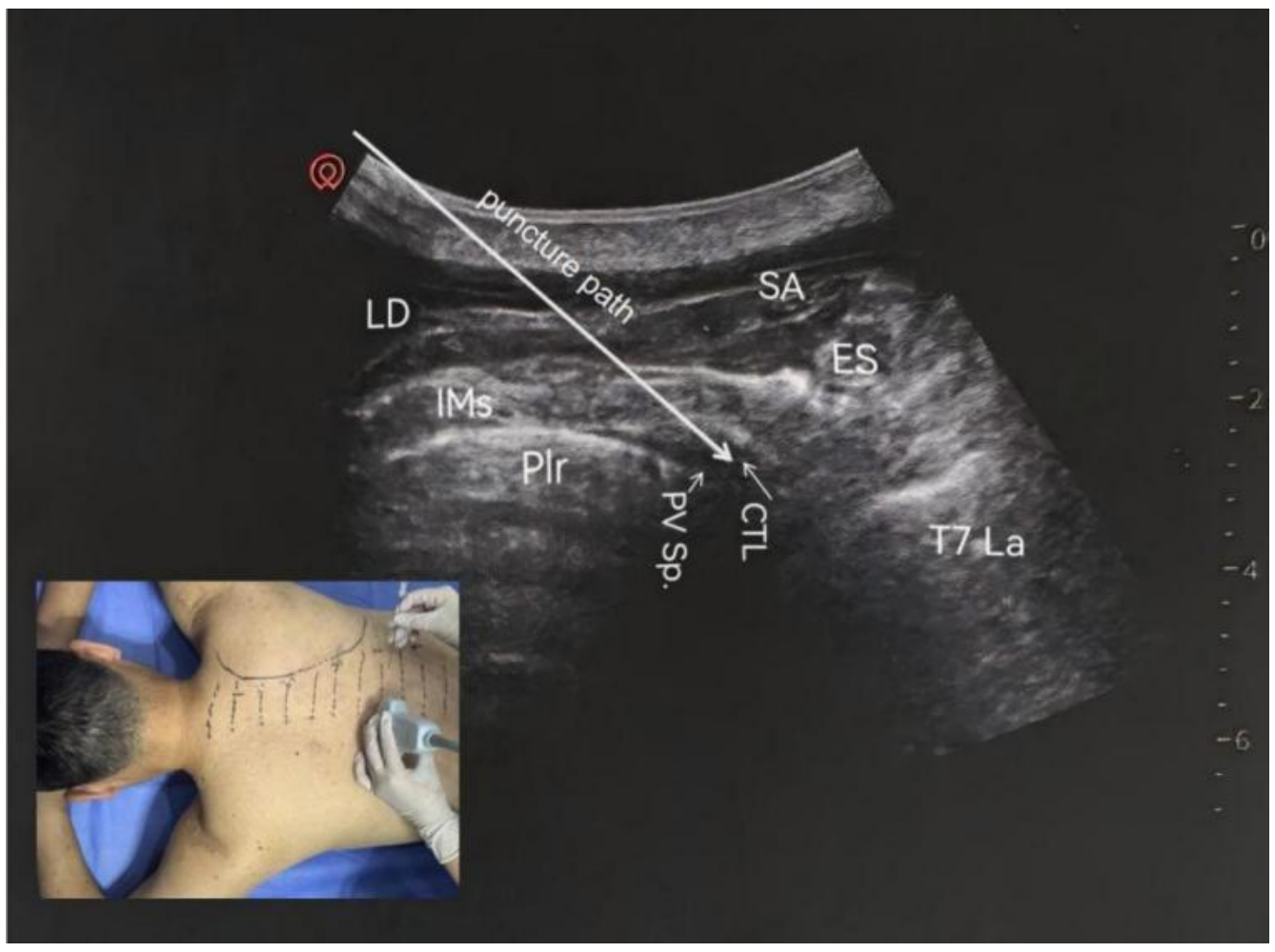

Figure S4. Puncture pathway for the T7 spinal nerve root.

LD = Latissimus Dorsi; IMs = Intercostal Muscles; Plr = Pleura; PV Sp = Paravertebral Space; CLT =Costotransverse Ligament; T7 La = T7 Lamina; ES = Erector spinae; SA = Serratus Anterior

Puncture procedure: The ultrasound probe was placed transversely at the T7-8 intervertebral foramen plane. The RF cannula was advanced to the lateral edge of T7 La using the short-axis plane technique with a strict medial needle trajectory to prevent pleural injury and pneumothorax.

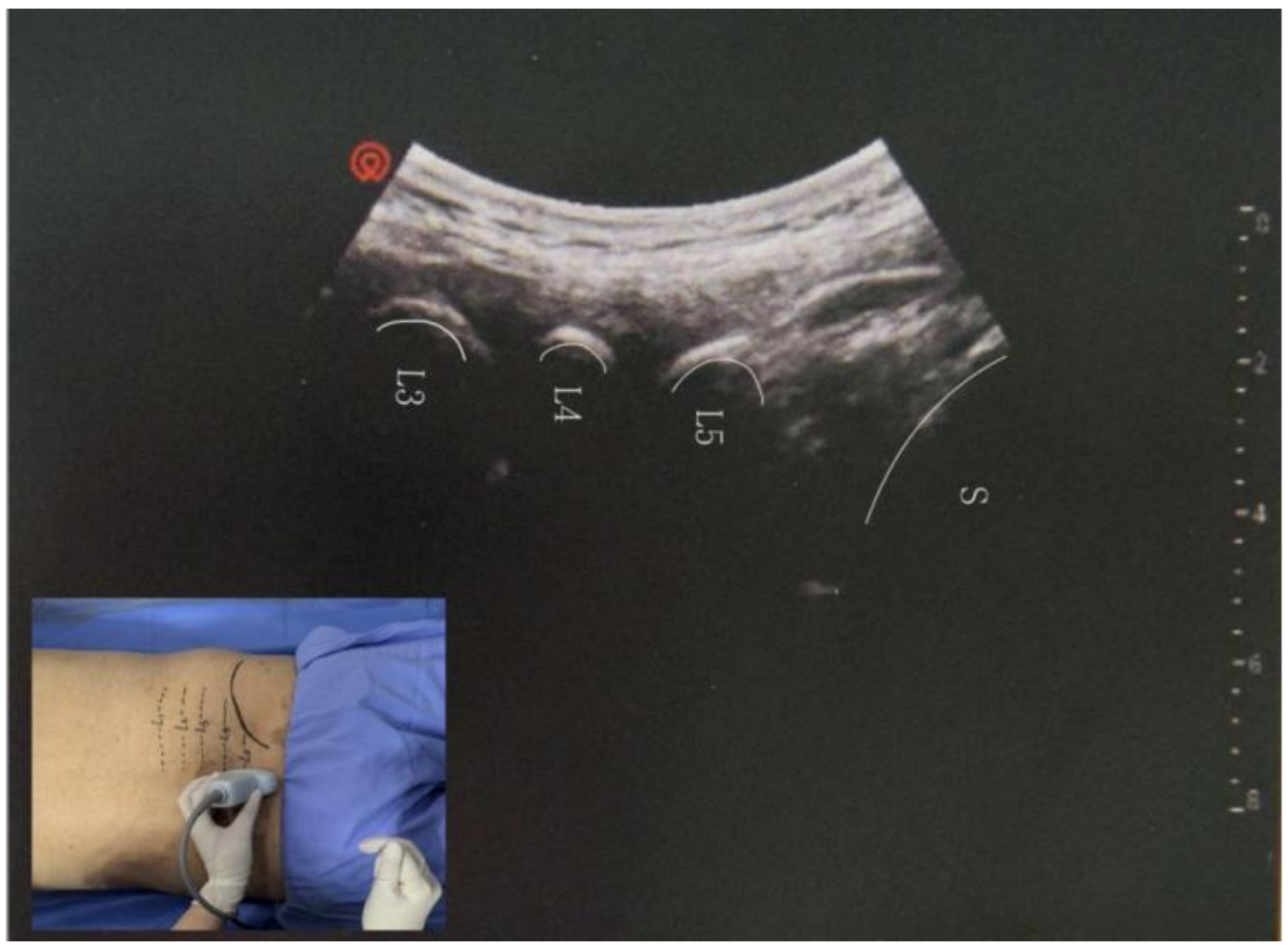

Figure S5. Longitudinal ultrasound landmarking for lumbar spinal nerve root localization.

Sacrum: L3 = 3rd Lumbar Vertebral Spinous Process; L4 = 4th Lumbar Vertebral Spinous Process; L5 = 5th Lumbar Vertebral Spinous Process.

Localization procedure: Patients were placed in the prone position with a thin pillow under the abdomen. A convex ultrasound probe (2-5 MHz, Sonosite M-Turbo) was placed longitudinally along the sacral midline, slid cranially to identify lumbar spinous processes (peak-like hyperechoic images) and transverse processes (discontinuous hyperechoic images). The transverse process closest to the sacrum was L5 TP; target lumbar segments (L1-L5) were confirmed by counting cranially

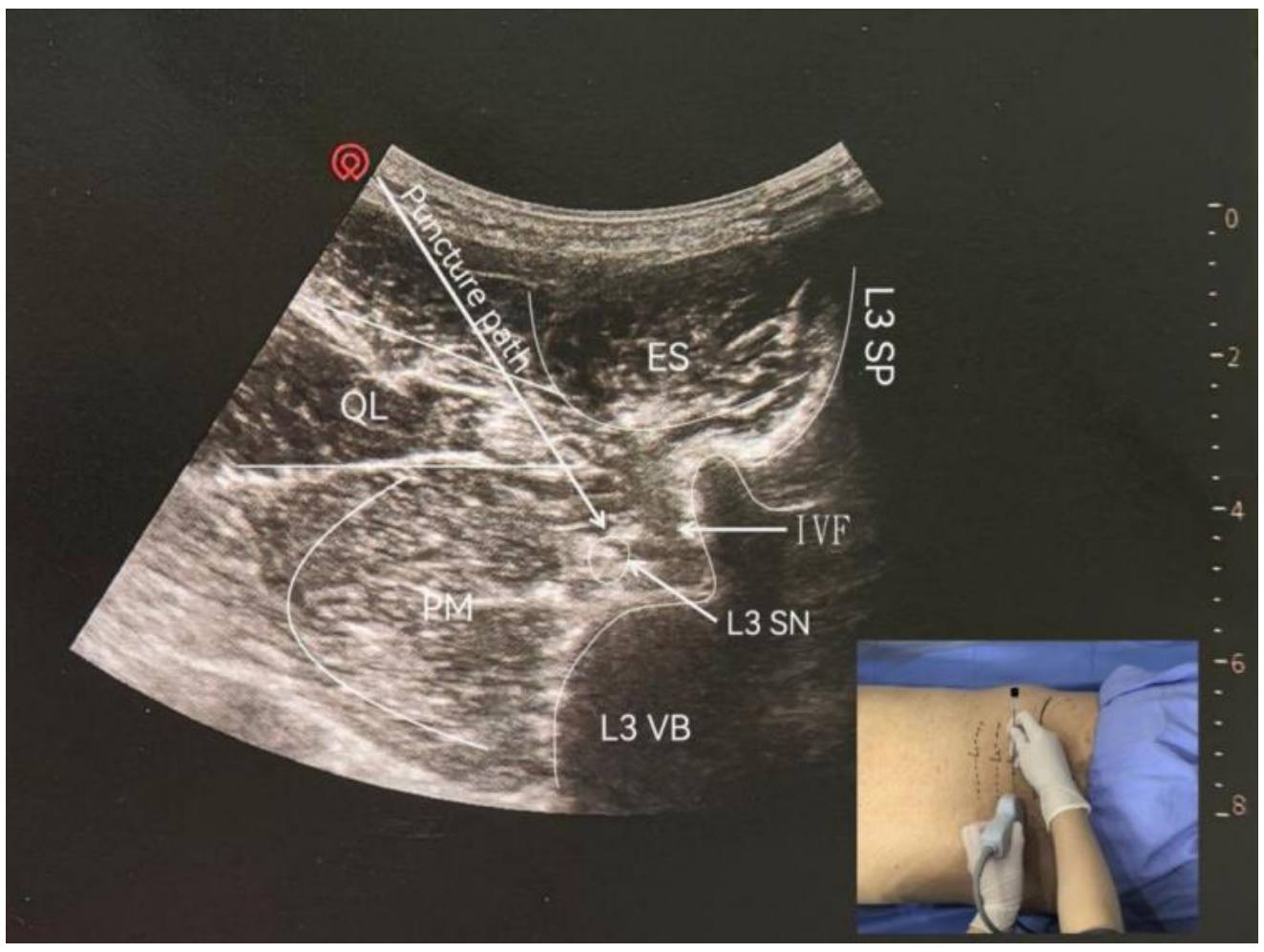

Figure S6. Puncture trajectory targeting the L3 spinal nerve root.

QL = Quadratus lumborum; PM = Psoas major; L3 VB = 3rd Lumbar Vertebral body; L3 SN = 3rd Lumbar spinal nerve; IVF = Intervertebral foramen; L3 SP = 3rd Lumbar Spinous Process; ES = Erector spinae; L3 La = L3 lamina.

Puncture procedure: After locating L3 transverse process, the probe was rotated transversely and slid caudally until transverse process disappeared. The depression between L3 La and L3 VB indicates L3-4 IVF. The RF cannula was advanced through ES and PM muscles to the vicinity of L3-4 IVF under ultrasound guidance.
